# Efficacy and safety of piezocision in accelerating maxillary anterior teeth en-masse retraction: study protocol for a randomized controlled trial

**DOI:** 10.1186/s13063-022-06389-4

**Published:** 2022-06-07

**Authors:** Yichen Xu, Liming Yu, Xianqin Tong, Yuhui Wang, Yuanyuan Li, Jie Pan, Yanjing Yang, Yuehua Liu

**Affiliations:** 1 Department of Orthodontics, Shanghai Stomatological Hospital & School of Stomatology, Fudan University, Shanghai, China; 2 Shanghai Key Laboratory of Craniomaxillofacial Development and Diseases, Fudan University, Shanghai, China; 3 Department of Pediatric Dentistry, Shanghai Stomatological Hospital & School of Stomatology, Fudan University, Shanghai, China

**Keywords:** Piezocision, En-masse retraction, Accelerate tooth movement, Orthodontic treatment, Randomized controlled trial/RCT

## Abstract

**Background:**

Orthodontic treatment is commonly more time-consuming in adults than in teenagers, especially when it comes to the maxillary en-masse retraction, which may take 9 months or even longer. As to solve this concern, orthodontists have been striving to seek new methods for shortening orthodontic treatment time. Piezocision, as a popular alternative treatment, has been widely used in different types of tooth movement. However, its effect on en-masse retraction of maxillary anterior teeth remains unclear. This randomized controlled trial intends to figure out the role piezocision plays in accelerating en-masse retraction.

**Methods:**

This protocol is designed for a prospective, single-center, assessor-blinded and parallel-group randomized controlled trial. Twenty adult patients aged from 18 to 40 whose orthodontic treatment required bilateral maxillary first premolars extraction will be randomly assigned to the piezocision group and the control group at a ratio of 1:1. The piezocision group will undergo en-masse retraction immediately after the piezo surgery, while the control group will start en-masse retraction directly. Both groups will be followed up every 2 weeks to maintain the retraction force until the end of space closure. The space closing time is set as the primary endpoint. Meanwhile, the secondary endpoints include the change of root length, labial and palatal alveolar bone thickness, vertical bone height, probing depth of maxillary anterior teeth, cephalometric measurements, visual analogue scale, and postoperative satisfaction questionnaire.

**Discussion:**

This study will attempt to provide more convincing evidence to verify whether piezocision will shorten the time of en-masse retraction or not. Distinguished with previous studies, our study has made some innovations in orthodontic procedure and primary outcome measurement, aiming to clarify the efficacy and safety of piezocision-assisted en-masse retraction in Chinese population.

**Trial registration:**

Chinese Clinical Trial Registry ChiCTR 1900024297. Registered on 5 July 2019

**Supplementary Information:**

The online version contains supplementary material available at 10.1186/s13063-022-06389-4.

## Administrative information

Note: the numbers in curly brackets in this protocol refer to SPIRIT checklist item numbers. The order of the items has been modified to group similar items (see http://www.equator-network.org/reporting-guidelines/spirit-2013-statement-defining-standard-protocol-items-for-clinical-trials/).

**Table:** 

Title {1}	Efficacy and safety of piezocision in accelerating maxillary anterior teeth en-masse retraction: study protocol for a randomized controlled trial
Trial registration {2a and 2b}.	Registry name: Accelerate orthodontic tooth movement by piezocision assisted orthodontics: a randomized controlled trial Trial identifier: ChiCTR1900024297
Protocol version {3}	Version 2.0 Issue date: 22 April 2019
Funding {4}	This trial was funded by the Science and Technology Commission of Shanghai Municipality (grant number 19411961900 and 20Y11904100), Shanghai Hospital Development Center (grant number SHDC12019126).
Author details {5a}	^1^Department of Orthodontics, Shanghai Stomatological Hospital & School of Stomatology, Fudan University ^2^Shanghai Key Laboratory of Craniomaxillofacial Development and Diseases, Fudan University ^3^Department of Pediatric Dentistry, Shanghai Stomatological Hospital & School of Stomatology, Fudan University
Name and contact information for the trial sponsor {5b}	1. Science and Technology Commission of Shanghai Municipality. Address: 200 People Avenue, Shanghai, China. Tel:(8621)-23111111 2. Shanghai Hospital Development Center. Address: No.2 Kangding Road, Shanghai, China. Tel:(8621)-96886
Role of sponsor {5c}	The funding sponsors will not participate in any procedures of the clinical trial, including study design, execution, data collection and statistical analysis.

## Introduction

### Background and rationale {6a}

To improve facial appearance and oral function, numerous people have an increasing demand for orthodontic treatment [1, 2]. One of the main challenges adult patients and orthodontists face is the prolonged orthodontic treatment time. It may take adults an average of 2–3 years to complete the treatments, depending on the severity of malocclusion and individual characteristics, much longer than the time required for adolescents [3].

The prevalence of convex deformity appeared relatively high in Chinese population. To correct this deformity, the most effective orthodontic treatment is extraction of bilateral maxillary first premolars, following with en-masse retraction (ER) of anterior teeth to improve the facial profile. It might take almost 9 months to retract anterior teeth in cases with upper premolars extraction assisted by miniscrew anchorage [4, 5]. Moreover, various complications, including caries, periodontal diseases, and root resorption, are largely attributed to the prolonged treatment time [6]. Therefore, accelerating orthodontic tooth movement (OTM) to shorten the orthodontic treatment time is currently one of the hottest research focuses.

A large amount of research has been conducted to dig diverse approaches out to accelerate tooth movement during the orthodontic treatment, consisting of surgical and non-surgical interventions generally. Surgical interventions contain corticotomy [7–9], distraction osteogenesis [10–12], and circumferential supracrestal fiberotomy [13–15], while nonsurgical ones include laser [16–21], vibration [22–25], drug [26, 27], ultrasound [28, 29], and electromagnetic therapy [30, 31].

However, non-surgical interventions have problems such as uncertain effects, short effective time, operating inconvenience, and high compliance requirements for patients [32, 33]. On the other hand, surgical interventions are increasingly accepted by both orthodontists and patients. Among them, corticotomy has received extensive attention in recent years [34].

Literature on corticotomy was initially put forward by Kole in 1959, in which corticotomy was used to form free bone blocks to accelerate OTM, resulting in great trauma [35]. Vercellotti and Podesta performed corticotomy with conventional periodontal flap and ultrasound osteotome for accelerating OTM since 2007 [36]. Dibart et al. reported a flapless approach in 2009, which was named after piezocision [8].

The corticotomy with a piezoelectric apparatus is performed after an interdental gingival incision. This method causes much less tissue traumatic damage to patients and accomplishes better prognostic quality, though it takes more time to realize the acceleration of OTM than the conventional flap surgery according to related literatures [37, 38]. However, the total period was shortened by 59% in the experimental group as piezocision was applied to alleviate the crowding of the mandibular dentition [39].

### Objectives {7}

The purpose of this trial is to investigate the efficacy and safety of piezocision in accelerating maxillary anterior teeth ER in normodivergent adults with convex deformity, whose treatment plans involve bilateral maxillary first premolars extraction.

### Trial design {8}

**Fig. 1:**
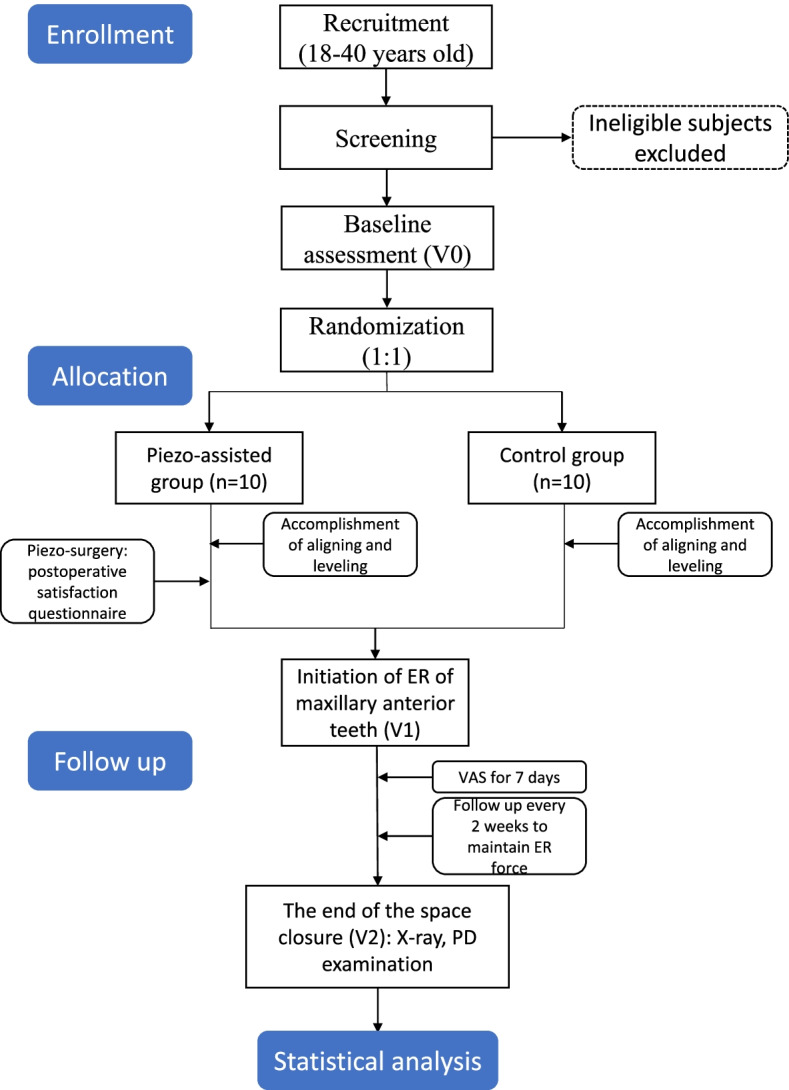
Flow chart of the trial design

This study will be a single-centered, assessor-blinded, and prospective randomized controlled trial (RCT), which was designed following the guideline of SPIRIT 2013 statement [40]. After initial screening and informed consent forms (ICF) obtained, all subjects will be randomized according to random numbers into the piecocision group (PG) and the control group (CG) in a 1:1 ratio. The observation period of this study will be 12 months. The study design is fully illustrated in the flowchart (Fig. 1).

## Methods: participants, interventions, and outcomes

### Study setting {9}

All the subjects who are eligible for the trial will be enrolled at Shanghai Stomatological Hospital, from October 2019 to March 2022. Shanghai Stomatological Hospital, as a municipal tertiary hospital, has set the relevant clinical departments of periodontitis and orthodontics, which can meet the demand of this trial. The recruiting announcements will be posted on the official websites of the hospital. In the preliminary trial stage, we have confirmed the scientific validity of piezocision surgical scheme through discussions with several periodontal and orthodontic experts, combined with the results of Dibart et al. [8].

### Eligibility criteria {10}

Participants can only be included in the study if the following criteria are met:

#### Inclusion criteria

1. Age from 18 to 40, male and female
2. Good oral condition: healthy periodontal condition or mild periodontitis (alveolar bone absorption less than 10%), no periapical infection, and unfilled caries
3. Convex deformity and normodivergent facial pattern with the treatment plan including 2 maxillary first premolars extraction
4. No or mild overcrowding in maxilla dentition

#### Exclusion criteria

1. History of temporomandibular disorders, systematic diseases, and systemic metabolic diseases
2. Laser, radiation workers
3. The presence of bone island, osteoma, apical cyst, root resorption, and root exposure in the tooth movement area
4. Periodontitis patients with alveolar bone absorption more than 10%
5. Gingival recession over 2 mm
6. Smokers
7. Abnormal bone metabolism (including taking anti-bone absorption drugs, hormone drugs, immunosuppressant)
8. Mental disorders or movement disorders
9. Pregnancy

### Who will take informed consent? {26a}

A qualified researcher will explain all the details about this trial, including the study procedure, benefits, and potential risks to all the subjects, and ensure that subjects are fully understood. Subsequently, informed consents will be obtained.

### Additional consent provisions for collection and use of participant data and biological specimens {26b}

Since this study will not involve the collection or use of biological specimens, this item is not applicable.

### Interventions

#### Explanation for the choice of comparators {6b}

Considering there is no internationally accepted treatment of accelerating OTM and the intervention in this study is a surgical one, we set the conventional orthodontic group as the control group, aiming to clarify the effectiveness and safety of piezocision on accelerating ER. In order to minimize the bias, participants in both groups will undergo the same orthodontic treatment procedure, operated by one senior orthodontist.

#### Intervention description {11a}

Following randomization, all the participants will receive straight wire orthodontic treatment utilizing self-ligating brackets (H20, Jinhua, Zhejiang, China) with the slot size of 0.020×0.028 inches. The maxillary dental arch will be aligned and leveled utilizing nickel-titanium wires in the following order: 0.014niti, 0.018niti, 0.014×025niti, and 0.018×025niti, with a senior orthodontist evaluating whether the alignment and leveling have been fully achieved. Subsequently, elastics with a force of 250 g per side will be applied to the traction hook on the distal side of the lateral incisor brackets, which is 8 mm long, with orthodontic anchorage provided by mini-screws implanted in the zygomatic alveolar crest area. Before beginning ER, the 0.018×025 stainless-steel wire should be left in place for 4 weeks to guarantee minimal sliding resistance.

The participants will have either piezo-assisted ER or conventional ER. Prior to retraction, patients in PG will get piezo surgery. Afterwards, all the participants must be revisited every 2 weeks to replace the elastics in order to maintain the retraction force until the space is completely closed. The piezocision operation will only be performed by a senior periodontist with clinical experience more than 10 years. Meanwhile, a chief orthodontist will be responsible for orthodontic treatment and mini-screws placement in the zygomatic alveolar ridge. Preceding the trial, all researchers will get training in accordance with the protocol. Subjects’ enrollment and assignment, standard treatment procedure, case report forms (CRF) filling, and adverse event reporting system (AERS) will be all elaborated in the training conference.

##### Piezocision group (PG)

Piezocision will be performed in 4 weeks after the placement of 0.018×0.025-in. stainless steel archwire and insertion of mini-screws. The patient will be anesthetized with local infiltration (Articaine 4% with 100,000 epinephrine) after a 1-min flush with chlorhexidine gluconate 0.12%. The surgical procedure will be performed as described by Dibart et al. [8]. Once anesthesia has set, vertical incisions will be performed in the attached gingiva of maxillary anterior region buccally and interproximally using a scalpel with a blade No. 15. Incisions will be started 4 mm below the base of the interproximal papillae to prevent any further gingival recessions. A piezo surgical knife (Satelec, Acteongroupe, Merignac, France) will be used to create the cut. The vertical length will be 5–8 mm and the depth of the cut will be approximately 3 mm. The incisions will not be sutured. Patients will be required to avoid hot and spicy food for 5–7 days and maintain oral hygiene after surgery.

**Fig. 2:**
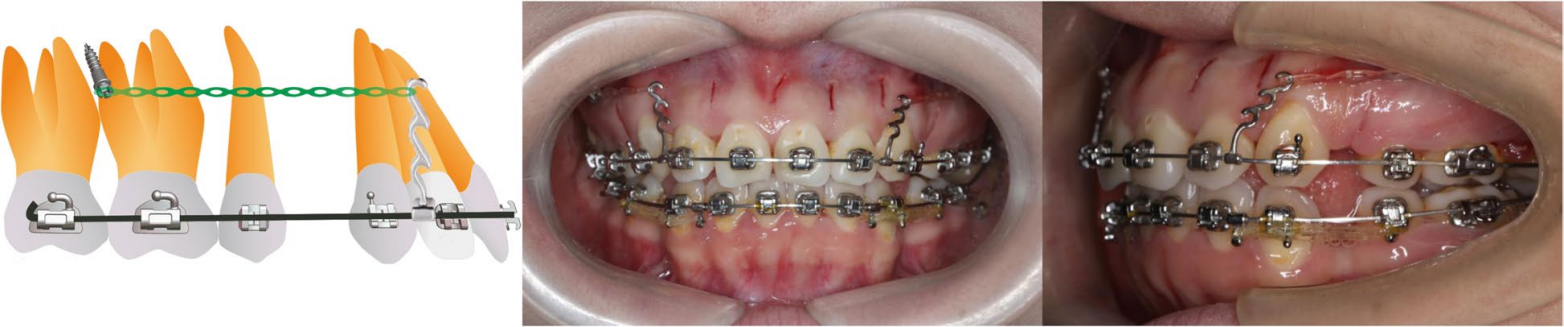
The ER treatment after the piezocision surgery

The elastic chain will be extended bilaterally from the mini-screws to the power hooks to initiate the ER immediately following the surgery. In order to confirm the force (250 g), a dynamometer (Xihu Cooperation, Hangzhou, China) will be used (Fig. 2).

##### Control group (CG)

This group of patients will only undergo orthodontic therapy. The elastic chain will be stretched from the screws’ head to the power hooks for ER.

#### Criteria for discontinuing or modifying allocated interventions {11b}

Subjects who are unable to complete follow-up for any reason can quit the study at any time without consequences. Participants can drop out of the trial if they have any of the following behaviors that affect outcomes during the trial, including smoking, pregnancy, and taking medications that may affect bone metabolism. Furthermore, if adverse events (AE) occur during treatment, the subjects will be allowed to discontinue the study. We will document a detailed record of the procedures.

#### Strategies to improve adherence to interventions {11c}

A clinical research nurse will organize outpatient visits and follow-up with participants in this research. Patients will get text messages reminding them of the appointment time in advance of the subsequent visits. When a patient fails to return on time, the research nurse will contact them through phone or WeChat. To further alleviate the stress on subjects, we will streamline the trial procedure to improve the efficiency of treatment.

#### Relevant concomitant care permitted or prohibited during the trial {11d}

Other therapies that could have an impact on the study’s outcomes, such as medications affecting bone metabolism or treatments which may facilitate OTM, will be prohibited during the period of the trial.

#### Provisions for post-trial care {30}

Participants will be informed that it is voluntary to be enrolled in this trial prior to the study. Subjects can refuse to participate or drop out of the study at any phase of the trial for any reason. Their medical care and rights will not be affected. We will record it faithfully. If an intervention-related adverse event happens during the trial, patients will get appropriate treatment measures for free and financial compensation after the evaluation of the expert committee.

### Outcome measures {12}

Outcome measurements will be recorded before randomization (V0), at the initiation of ER (V1) and after space closure (V2). The primary outcome is the space closing time, which can reflect the effectiveness of piezocision directly. The secondary outcomes are designed to compare the periodontal health, the alveolar bone changes, and torque control of maxillary anterior teeth before and after ER between the two groups. We will also pay attention to the patient-centered outcomes as to evaluate the surgery more comprehensively.

#### Primary outcome

The primary outcome with respect to the efficiency of piezo surgery is the space closing time (T_V2-V1_) from the initiation of ER (V1) to the end of the space closure (V2). All the subjects will be revisited every 2 weeks. An assessor blinded to groups assignment will take charge of recording each visit time and evaluate whether the extraction space has been completely closed by using dental floss to confirm the contact point of adjacent teeth.

#### Secondary outcomes

The secondary outcomes will contain the root length, thickness of labial and palatal alveolar bone, vertical bone height (VBH), probing depth (PD) of maxillary anterior teeth, cephalometric analysis, visual analogue scale (VAS), and postoperative satisfaction questionnaire (PSQ).

#### Root length

**Fig. 3:**
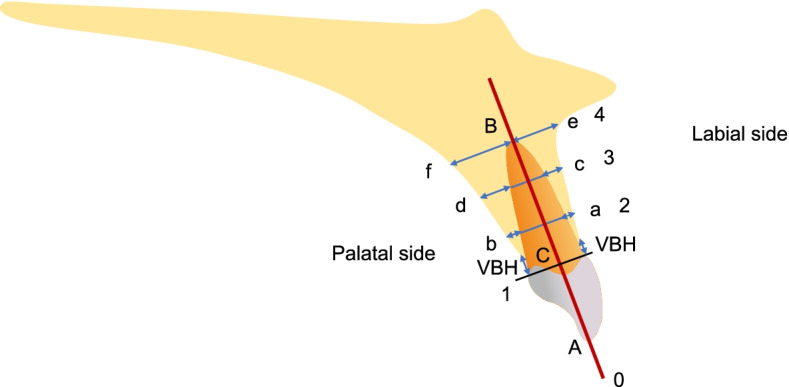
Measurements of the root length, labial and palatal alveolar bone thickness, and the vertical bone height of maxillary anterior teeth. Line 0 represents the long axis of the teeth and line 1 the CEJ, while line 4 is parallel to line 1 and passes through the root apex. The root length will be divided into 3 equal parts by line 2 and line 3. Meanwhile, a, b, c, d, e, and f represent the distance from the labial and palatal alveolar bone to the root surface. The VBH will be measured as the distance between the CEJ and the alveolar bone crest

Participants will take cone beam computed tomography (CBCT) scans at V0 and V2 using KaVo 3D eXam (KaVo, Biberach, Germany). The CBCT scan data will be stored as Digital Imaging and Communications in Medicine (DICOM) files and imported into a software (OnDemand3D Application, CyberMed Corporation, Seoul, Korea) for reconstruction and measurement. The root length will be measured as the vertical distance from the cementoenamel junction (CEJ) to the root apex (Fig. 3).

#### Thickness of labial and palatal alveolar bone

Paralleled to and passing through the long axis of each examined tooth, the sagittal plane will be adjusted to be perpendicular to the labial surface of the tooth in multiplanar reconstruction image. The root length will be divided into 3 equal parts (L0 representing the long axis of the teeth; L1 representing the CEJ; L4 representing the root apex). The thickness of the labial and palatal alveolar bone of the anterior teeth will be measured from the cortical plate of the alveolar bone to the root surface (Fig. 3).

#### VBH

The VBH is defined as the perpendicular distance between the alveolar ridge crest and the CEJ, which will be measured in the same sagittal plane mentioned above (Fig. 2).

#### PD

The probing depth of maxillary anterior teeth will be measured using the electronic Florida probe (Florida Probe Cooperation, Gainesville, Florida, USA) with the controlled force of 15 g.

#### Cephalometric measurements

**Fig. 4:**
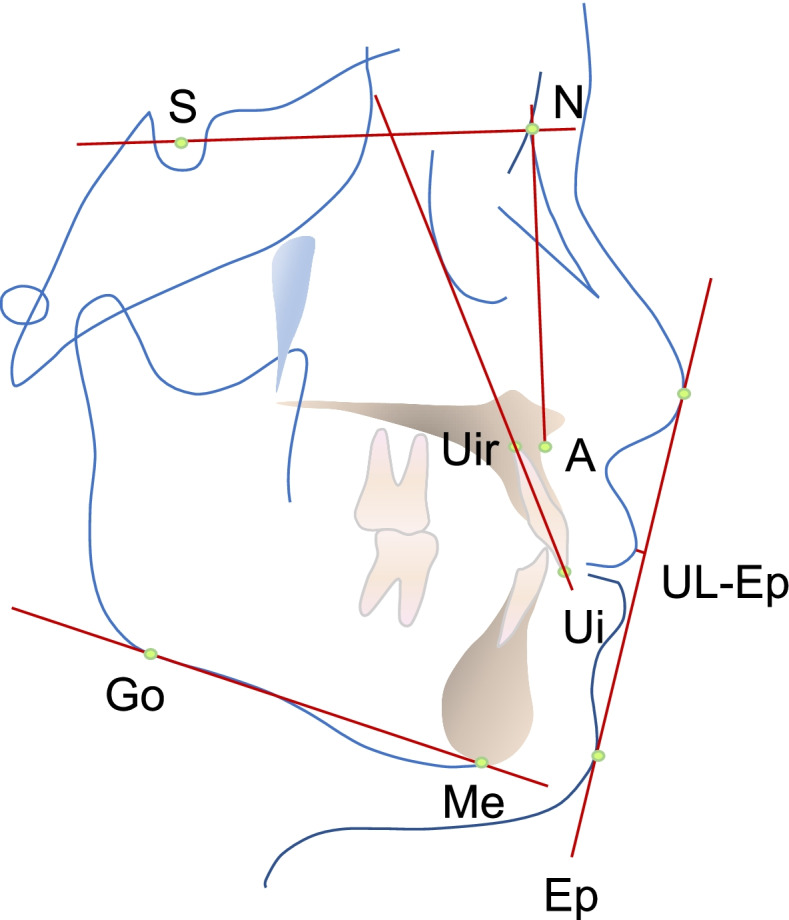
Cephalometric landmarks and indicators. *S* sella, *N* nasion, *A* subspinale, *Ui* upper incisor, *Uir* upper incisor root, *Me* menton, *Go* gonion, *UL* upper lip anterior, *MP* the mandibular plane, *Ep* the aesthetic plane, *UL-Ep* distance between UL and the aesthetic plane

Preoperative and postoperative lateral radiographs of each subject will be all taken by the same X-ray machine (OC200D, Ingram, Finland). The Dolphin Software (Dolphin Imaging Software V11.7, USA) will be used for cephalometric analysis of the lateral radiographs. Cephalometric parameters are shown in Fig. 4, including SNA, an indicator reflecting the anteroposterior position of the maxillary bone relative to the cranium; MP-SN, the index reflecting the steepness of mandibular body; U1-SN, the index reflecting the inclination of maxillary anterior teeth; and UL-Ep, an indicator of the soft tissue protrusion of the upper lip.

#### VAS

The level of discomfort will be evaluated utilizing VAS from the day of ER initiation (V1) in both groups for successive 7 days. Briefly, we will grade the intensity of pain on a scale from 0 (no pain) to 10 (maximum pain).

#### PSQ

PSQ will be filled out on the day of surgery for subjects in PG. Patients will share their subjective perception during the surgical treatment, including the feeling of vibration, sound fear, pain, teeth ache, and teeth numbness, which will be graded on a scale from 0 (the minimum of feeling) to 5 (the maximum of feeling).

### Participant timeline {13}

The timeline of the clinical trial is shown in Table 1.

**Table 1:**
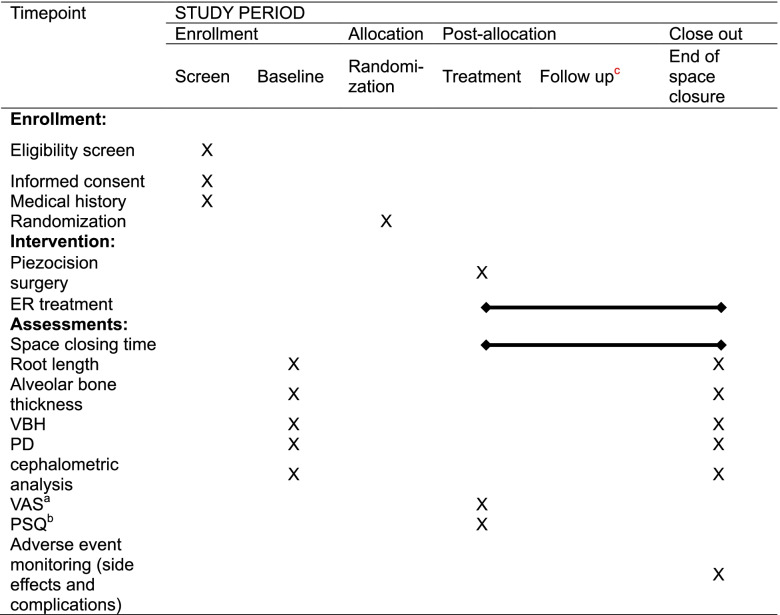
Schedule of enrollment, interventions, and assessments (recommended by SPIRIT)

*VBH* vertical bone height, *PD* probing depth, *VAS* visual analogue scale, *PSQ* postoperative satisfaction questionnaire

^a^VAS will be filled out daily for 7 days postoperatively

^b^PSQ will only be filled out in participants of piezocision group

^c^All the subjects will be followed up every 2 weeks. The status of space closure will be monitored every visit time

### Sample size {14}

The sample size will be calculated using PASS system (version 15.0.5) in this study. According to a study reported in 2016, the mean alignment time is reduced by 40% in the intervention group compared to the control group [41]. Our anticipated hypothesis is that the overall treatment time of ER in CG is assumed to be 9 months, while in PG is 5.4 months, with standard deviation of 2 for both groups. Assuming equal variance, 8 participants in each group can provide 90% power to reject the null hypothesis. The significance level is 0.05 and the power is 90%. Considering an anticipated 20% dropout rate, the final sample size is a total of 20, and 10 participants in each group.

### Recruitment {15}

Shanghai Stomatological Hospital will serve as the primary research center for participants’ recruitment. Eligible subjects will be recruited through posters in the waiting area and recruitment ads on official website and WeChat official account of the hospital, from October 2019 to March 2022. Shanghai Stomatological Hospital comprises relevant departments involved in this study, including orthodontic department and periodontal department, with a team of medical professionals. Eligible patients from outpatient clinics will be informed by clinical physicians and contacted by clinical research coordinators (CRC) to arrange screening visits. After obtaining the informed consent, screening will be conducted, and subjects who meet all inclusion and exclusion criteria will be enrolled in the study.

## Assignment of interventions: allocation

### Sequence generation {16a}

An independent statistician will generate a random sequence of 40 sequential numbers utilizing SPSS (version 24.0).

### Concealment mechanism {16b}

Sequential numbered, opaque, sealed envelopes (SNOSE) will be used as a method of allocation concealment.

### Implementation {16c}

An independent researcher will be responsible for the enrollment of participants. After signing the ICF and screening, eligible subjects will receive SNOSE with orderly numbers according to the order of enrollment. The investigator will write down the patients’ detailed information (name, gender, age, and ID number) on the SNOSE and take out the cards immediately. The subjects’ group will be determined by the random numbers in the SNOSE. The random operator and the random date will be recorded.

## Assignment of interventions: blinding

### Who will be blinded? {17a}

Since the intervention in this RCT is a surgery treatment and setting a sham surgery may cause unnecessary harm to the participants, the medical staff and subjects cannot be blinded in this study. To minimize the bias, the outcome assessor and data statistical analyst will be blinded during the trial.

### Procedure for unblinding if needed {17b}

Since the researchers and participants in this study will not be blinded, the procedure for unblinding is not applicable in this trial.

## Data collection and management

### Plans for assessment and collection of outcomes {18a}

After randomization, participants in PG will undergo ER immediately after piezocision surgery, while the ones in CG will receive ER therapy directly. All of them will be asked to fill out the VAS for 7 successive days after ER to compare the discomfort feeling between the two groups. Subjects in PG will additionally fill in the PSQ after the surgery.

The primary outcome is the space closing time (T_V2-V1_), which will be measured from the initiation of ER (V1) till the end of the space closure (V2).CBCT will be taken at V0 and V2 to measure the root length and the thickness of the labial and palatal bone of the 6 maxillary anterior teeth. Dolphin will be used for cephalometric analysis of lateral radiographs to analyze indexes related to dental, skeletal, and soft tissue changes including U1-SN, SNA, MP-SN, and UL-E.

Study staff, including orthodontists, periodontists, clinical study nurses, CRC, assessors, and statisticians, will be trained in data management.

### Plans to promote participant retention and complete follow-up {18b}

Patients will get text messages reminding them of the appointment time in advance of the visits. When a patient fails to return on time, a clinical study nurse will contact them through telephone or WeChat. We will streamline the trial procedure to improve the efficiency of treatment.

### Data management {19}

This study uses printed CRF and network-based electronic case report forms for transmission of study information and data. The CRC will fill in the CRF with original data and input them into the electronic data capture (EDC) system. A clinical research assistant will verify the integrity and precision of the data. Only investigators and authorized personnel who know the username and password can get access to the EDC to view the subject’s information. All manual (paper) and electronic data will be kept at the sponsor center for 5 years after publication.

### Confidentiality {27}

Subjects’ name, ID card, telephone, address, and other personal information will be treated anonymously to protect personal privacy. Unauthorized access to clinical trial data will not be permitted except investigators, clinical research center staff, ethics committee members, monitors or auditors. After the trial, clinical trial documents will be uniformly stored in the archive’s office of the institution. Only the members of the original clinical trial research team and inspectors are eligible to view the documents with the approval of the principal investigator (PI) and the director of the clinical research center.

### Plans for collection, laboratory evaluation, and storage of biological specimens for genetic or molecular analysis in this trial/future use {33}

Since biological specimens are not involved in this study, this item is not applicable.

## Statistical methods

### Statistical methods for primary and secondary outcomes {20a}

All data will be analyzed by an independent statistician utilizing SPSS (Version 24.0). The data analysis set in this study will comprise of the full analysis set (FAS), per protocol (PP) set, and safety set (SS). The subjects who receive the treatment at least once after randomization and have corresponding evaluation will be analyzed as the FAS. The subjects who follow the protocol strictly and complete the follow-up will be enrolled in the PP set. SS includes the subjects who have received intervention at least once with safety assessment during the trial. The primary endpoint will be analyzed in the FAS and PP, and secondary endpoints will be analyzed in the FAS. The safety of the treatment will be analyzed in the SS.

Continuous data will be presented as the mean ± standard deviation (SD) or median (P25-P75), while categorical data will be presented as absolute frequencies (percentages). *T* test or Mann-Whitney *U* test will be used to compare primary endpoints between groups. For secondary endpoints, the *t* test or Mann-Whitney *U* test will be used to compare continuous variables between groups, and categorical variables will be analyzed by chi-square test or Fisher’s exact test. The statistical tests used in the study will be two-sided. When *P*<0.05, it will be considered statistically significant.

### Interim analyses {21b}

No interim analyses will be planned in this study.

### Methods for additional analyses (e.g., subgroup analyses) {20b}

No subgroup analyses or any other additional analyses will be planned in this study.

### Methods in analysis in handle protocol non-adherence and any statistical methods to handle missing data {20c}

For subjects who drop out of the study halfway or have not completed ER at 12 months, the primary outcome will be filled out for 12 months.

### Plans to give access to the full protocol, participant-level data, and statistical code {31c}

We will publish the trial protocol in a peer-reviewed journal. Original data will be stored in the de-privacy processed will be stored in the database managed by the data management platform of Shanghai Stomatological Hospital. The data can be reasonably required with the approval of PI.

## Oversight and monitoring

### Composition of the coordinating center and trial steering committee {5d}

This study is a single-centered trial, which will be supervised by clinical research center and ethics committee. The quality control of this trial will be ensured by an independent quality control coordinator (QCC) and a clinical research associate (CRA) in accordance with the principal of Good Clinical Practice (GCP). They will take charge of monitoring the study progress, checking whether the protocol is followed, and submitting the study status and revision suggestions to PI. PI will be responsible for organizing discussions on study protocol, revisions, and annual reports to the Ethics Committee. The data management team will include PI, two orthodontists and a data management (DM) team for CRF design, CBCT measurements, and cephalometric analysis. The DM team will confirm the integrity, accuracy, and integrity of data collection, entry, and analysis, as well as maintaining the database.

### Composition of the data monitoring committee, its role, and reporting structure {21a}

The intervention in this study is low risk with few safety concerns. In addition, assessors and CRC will be trained to collect and input data in strict accordance with protocol requirements. Reliable methods have been established for data measurements. Therefore, no data monitoring committee will be set up in this trial.

### Adverse event reporting and harms {22}

All AE will be recorded in detail on the CRF by researchers, including the time of starting and ending, grade, and relationship with the intervention treatment. The outcome and progress of AE will be closely monitored. AE will be assessed and managed by a supervisory team of 3 experienced dentists. All patients with AE should be followed up not only during the clinical trial, but after the end of the trial, until it is completely stabilized. The follow-up data will be recorded in CRF. Subjects who drop out of the study due to AE will also be followed up to record relevant data. Serious adverse event (SAE) will be reported to relevant health administrative department and the Ethics committee within 24 h. All participants can quit the trial at any time. Circumstances under which participants may discontinue in this study include the occurrence of AE, SAE, and the discovery of evidence that might affect the study results.

### Frequency and plans for auditing trial conduct {23}

This study is initiated by investigators. PI will hold regular meetings every 6 months to check the project. The audit includes original medical records, CRF completion, adverse events, and ICF. PI will submit an annual report on research progress to the Ethics Committee.

## Plans for communicating important protocol

### Amendments to relevant parties (e.g., trial participants, ethical committees) {25}

Any modification of the protocol will be submitted to the Ethics Committee for approval. The new version of the documents will be uploaded to the Chinese Clinical Trial Registry for updating. We will keep detailed records of all the procedures.

## Dissemination {31a}

The final research report will be published in a peer-reviewed journal. The results will be presented to the public at international conferences and seminars.

## Discussion

Convex deformity has always been a problem that concerns some Chinese adults a lot [42, 43]. Adult patients with convex deformity, mainly aged from 18 to 40, have high treatment expectations, demanding the most retraction to achieve a straight profile [44, 45]. The typical orthodontic treatment is the anterior teeth retraction after the extraction of first premolars [46]. Two basic bio-mechanical strategies can be used to close extraction spaces: two-step retraction (TSR) or ER. It is reported that TSR takes between 1.8 and 2.2 times longer than ER to close the extraction spaces, so that ER is commonly used for anterior teeth retraction in clinical practice to improve the patients’ profile [47, 48]. However, the duration of ER treatment depends on the rate of alveolar bone remodeling and the tissue response of adults to orthodontic forces which is less reactive compared to adolescent patients, resulting in longer orthodontic treatment time [49]. How to accelerate the tooth movement in adults is one of the hot topics in current studies, and it is necessary to find an effective way to accelerate ER treatment to shorten the orthodontic time of convex deformity [50].

As a minimally invasive method of OTM acceleration, piezocision has been widely used in clinical practice [51]. To the best of our knowledge, piezocision has been verified to have an acceleration effect on several types of tooth movement, including dentition alignment, canine distalization, molar intrusion, and protraction [41, 52–54]. However, there is still no clear conclusions on the efficacy of piezocision in accelerating ER treatment so far. Two recent trials studied the effectiveness of piezocision in accelerating ER in premolars extraction orthodontic cases reached disputed conclusions [55, 56]. Different trial designs and study populations are probably the main reasons accounting for the controversial conclusions. In this study, piezocision will be performed in the labial anterior region before ER. It is expected that the retraction time will be shortened by piezocision through accelerating bone remodeling so as to improve the efficiency of orthodontic treatment.

There are several differences between our study and previous study designs, as detailed below. Firstly, the amount of space closure in 4 months was mainly observed as a primary outcome in previous studies [55]. The primary outcome in this trial is the space closing time from the initiation of ER to the end of space closure. The observation time is longer than previous studies which can reflect the effects of piezocision in ER more directly. Secondly, the torque of anterior teeth could be better controlled by the orthodontic force applied by zygomatic alveolar ridge implant anchorage with 8-mm traction hook. The resistance center of anterior teeth is reported to be approximately 12.2 mm apically from the incisor edge and approximately 6.5 mm apical to the bracket position for the six-tooth units [57, 58]. The use of 8-mm traction hook can ensure that the force line can be vertically as close to the resistance center as possible so that the torque of anterior teeth could be better controlled. Besides, due to the position of zygomatic alveolar ridge, the direction of force applied by the anchorage screws implanted in this area assisting anterior tooth retraction will be closer to the resistance center, which is conducive to the bodily movement of the anterior teeth and prevents the anterior teeth from being too upright due to torque loss [59]. Thirdly, this trial will be conducted in Chinese population, which has not been reported previously.

This study also has some limitations. First of all, subjects and the piezocision operator cannot be blinded due to the nature of surgery. To reduce the research bias, participants in each group will be treated separately to avoid mutual communication and the assessor and statistician will be blinded. In addition, many factors may affect the efficiency of piezocision, including the length and depth of the surgical incision, as well as the magnitude and frequency of the orthodontic force, which will be explored in further research. Thirdly, this study is a single-centered trial, which may lead to restriction of future generalization and lack of external validity.

In summary, it has not reached an agreement on whether the piezo surgery will impose a positive acceleration effect on the maxillary anterior teeth ER treatment or not. This trial is expected to provide more reliable clinical evidence of the potential value of piezocision in this field.

## Trial status

The study is currently in the recruitment phase, which began in October 2019 and is expected to be completed by the end of March 2022. The protocol version is 2.0 (issue date 22 April 2019).

## Supplementary Information

**Additional file 1. **SPIRIT Checklist for *Trials*.

## Data Availability

The data can be reasonably required with the approval of corresponding author.

## Abbreviations

ER: En-masse retraction
OTM: Orthodontic tooth movement
RCT: Randomized controlled trial
ICF: Informed consent forms
PG: Piezocision group
CG: Control group
CRF: Case report forms
AERS: Adverse event reporting system
AE: Adverse events
VBH: Vertical bone height
PD: Probing depth
VAS: Visual analogue scale
PSQ: Postoperative satisfaction questionnaire
CBCT: Cone beam computed tomography
DICOM: Digital imaging and communications in medicine
CEJ: Cementoenamel junction
CRC: Clinical research coordinators
SNOSE: Sequential numbered, opaque, sealed envelopes
EDC: Electronic data capture
PI: Principal investigator
FAS: Full analysis set
PP: Per protocol
SS: Safety set
SD: Standard deviation
QCC: Quality control coordinator
CRA: Clinical research associate
GCP: Good clinical practice
DM: Data management
SAE: Serious adverse events
